# Detection of linear features including bone and skin areas in ultrasound images of joints

**DOI:** 10.7717/peerj.4411

**Published:** 2018-03-15

**Authors:** Artur Bąk, Jakub Segen, Kamil Wereszczyński, Pawel Mielnik, Marcin Fojcik, Marek Kulbacki

**Affiliations:** 1 Research & Development Center, Polish-Japanese Academy of Information Technology, Warsaw, Poland; 2 Institute of Informatics, Silesian University of Technology, Gliwice, Poland; 3 Department for Neurology, Rheumatology and Physical Medicine, Helse Førde, Førde, Norge; 4 Faculty of Engineering and Science, Western Norway University of Applied Sciences, Norway; 5 DIVE IN AI, Wroclaw, Poland

**Keywords:** Synovitis, Medical imaging, Machine learning, Linear detector

## Abstract

Identifying the separate parts in ultrasound images such as bone and skin plays a crucial role in the synovitis detection task. This paper presents a detector of bone and skin regions in the form of a classifier which is trained on a set of annotated images. Selected regions have labels: skin or bone or none. Feature vectors used by the classifier are assigned to image pixels as a result of passing the image through the bank of linear and nonlinear filters. The filters include Gaussian blurring filter, its first and second order derivatives, Laplacian as well as positive and negative threshold operations applied to the filtered images. We compared multiple supervised learning classifiers including Naive Bayes, k-Nearest Neighbour, Decision Trees, Random Forest, AdaBoost and Support Vector Machines (SVM) with various kernels, using four classification performance scores and computation time. The Random Forest classifier was selected for the final use, as it gives the best overall evaluation results.

## Introduction

Synovitis—that is, an inflammation of the synovial membrane around the joint—may lead to a dangerous and irreparable joint degeneration. It may cause swelling and pain in the relatively early stages, but its prolonged persistence may result in a functional disability. An often used noninvasive method of synovitis assessment is a doctor’s examination of ultrasound images of joints. Since such examination consumes time of a trained medical practitioner, and the results from different examiners may vary due to subjectivity of human judgment, it is desirable to develop an automated tool for the assessment of synovitis.

This paper describes a method for bone and skin detection which is a part of the system for automated assessment of synovitis activity, a work in progress within the Polish-Norwegian research project Medusa (Medusa, 2015). In the Medusa system, the bone and skin detection method provides the initial estimates of the bone and skin. The improved, final estimates (Segen, Kulbacki & Wereszczyński, 2015) are obtained by registering a model with a structural description of the image in the form of a group of parts that includes the results of the detector described here and an initial estimate of the joint which is described in a separate article (Wereszczyński et al., 2015).

The area of research that is nearest to the detection problem addressed in this paper is the analysis and processing of medical ultrasound images. This is explained by the shared characteristics of ultrasound image formation process, where the image is built as a plot of travel times of ultrasound pulses, that are sent by a transducer and received back after being reflected by organs and tissues at different depths. A noticeable feature of medical ultrasound images is the presence of speckle noise, and all ultrasound image analysis techniques must include some form of speckle noise reduction or removal. Among the accessible publications on analysis and processing of medical ultrasound images, the literature search conducted by the authors did not find articles related to the detection of skin or bone.

Yap, Edirisinghe & Bez (2007) proposed a fully automatic detection of breast lesion in ultrasound images. They used a histogram equalization, hybrid filtering, multifractal analysis and a rule-based approach for identification of region-of-interest, which is used as a seed-point in thresholding based background separation and followed by active contour method for final lesion boundary detection. Supriyanto et al. (2011) used a threshold segmentation and combination of morphological techniques for early detection of breast cancer and several other types of abnormalities in breast tissues. Lokesh, Shailaja & Nanda (2014) used automatic contouring and texture analysis for the classification of breast lesions. The anisotropic diffusion filtering is applied to remove the speckle noise while preserving the lesion boundaries. A segmentation based on watershed transform finds the contour of the breast lesion and features based on the histogram of co-occurring greyscale values are extracted from the segmented lesion and used in the SVM classifier to identify the breast lesion as benign or malignant. Chen et al. (2016) describe detection and segmentation of anatomical structures by multi-domain regularized deep learning method which uses a convolutional network and iteratively refines the results. The method is applied to obstetric and cardiac ultrasound images. Kisilev et al. (2013) construct a multi-stage learning of lesion-specific boundaries for automatic detection of breast lesion in ultrasound images. They apply multiple image filters and segmentation algorithms to obtain various textural characteristics and local directional coherence features from the image, which are used by an SVM classifier for automatic detection of breast lesion boundaries. Their approach similarly to the method proposed in this paper uses image filters as features for pixel classification, however our method is distinct in other aspects. It uses a different, unique set of image features, most of which are one-dimensional and include threshold operators, and a discrete optimization process is applied to find the best configuration of the features and filter parameters.

## Method Overview

**Figure 1:**
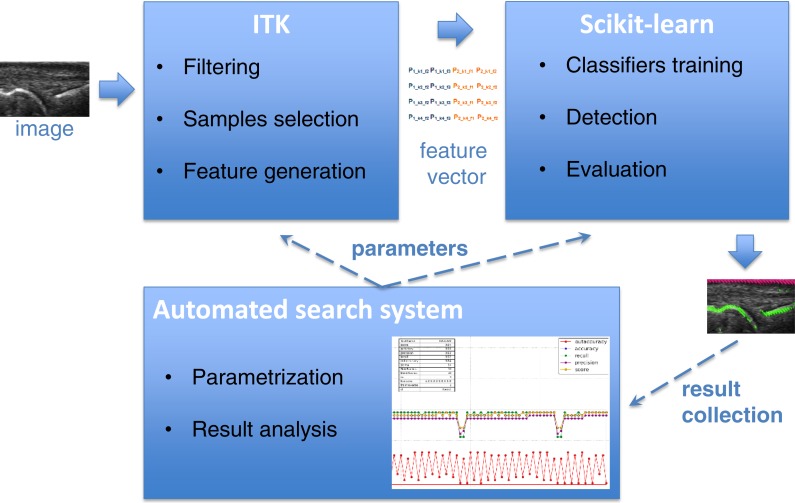
The architecture of system and operations flow between its components.

The main purpose of the detectors described in this paper is to find the bone and skin parts as lines in the image. Supervised learning accomplishes this by using training samples extracted from annotated areas of training images by filtering. The Insight Segmentation and Registration Toolkit (ITK) (Johnson et al., 2013) algorithms are used for image filtering including Gaussian smoothing, the first and second derivatives, Laplacian and threshold filters. The results of filtering are then applied to generate an appropriate vector of features used as training samples for learning algorithms. The scikit-learn tool (Pedregosa et al., 2011) was used for learning selected features, which is a specialized machine learning environment with many configurable classifiers. A few different classifiers were evaluated in this work including Gaussian Naive Bayes (Zhang, 2004), k-Nearest Neighbours (kNN) (Altman, 1992), Support Vector Machines (SVM) (Boser, Guyon & Vapnik, 1992; Chang & Lin, 2011), Decision Trees (Breiman et al., 1984; Hastie, Tibshirani & Friedman, 2009), Random Forest (Breiman, 2001) and AdaBoost (Freund & Schapire, 1997; Zhu et al., 2009). Both the generation of the feature vector in ITK and the learning algorithms in scikit-learn require a large number of parameters, which have a strong influence on the result of detection. Additionally, different combinations of filters applied to feature vector generation give significantly different learning efficiencies. It requires finding the optimal configuration of the system over the vast space of parameters. For that reason, the parametrization tool was developed for an automatic execution of feature extraction and learning procedure in an iterative way combined with the effective result visualization module. It allows executing a big number of experiments to find some optimal parameters as well as most efficient filter combination in much easier and quicker way than manual trials. The general architecture of bone and skin detector and the operations flow are illustrated in Fig. 1.

The following sections describe more details regarding the steps of feature extraction and applied learning and present the results of empirical evaluation.

Regional Committee for Medical and Health Research Ethics, Region West, Norway has approved the study (ref. 2013/743). All participants signed an informed consent form.

## Feature Extraction

Learning algorithms require appropriate representative features extracted from raw images to be able to learn and detect the bone and skin areas efficiently. The form of such features must allow representing different regions of interest in the image in a discriminative way.

The features in the determined form are computed in two parts of the method: (1) extraction of features from training images used as training data for learning and (2) extraction of features from the test image subjected to detection of bone and skin by already trained algorithms. Both cases require slightly different procedures of extraction, though the basic concept is similar. These procedures are described in the following sections.

### Features for training

**Figure 2:**
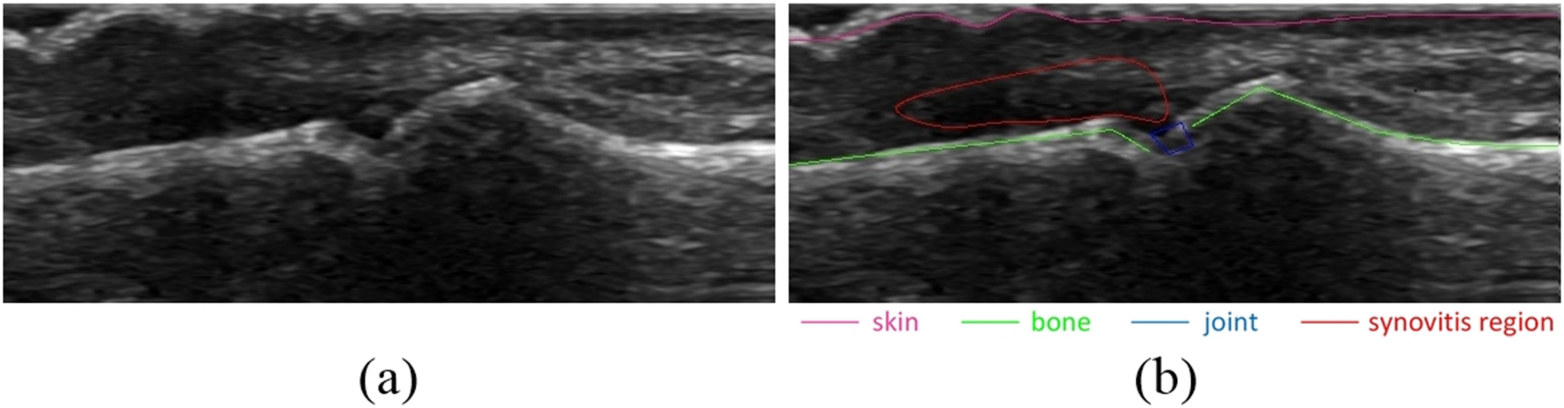
Example of image annotations for training and evaluation: (A) image without annotations, (B) image with annotations. Skin, bones, joint and synovitis region are marked with different colors.

As the bone and skin detector uses supervised learning, the training data for algorithms must explicitly indicate for each set of features extracted from the image, whether it is related to bone or skin or to neither of them. This information must be provided a priori as a set of annotations attached to each processed image of a training set. An example of image annotations is shown in Fig. 2B.

The pixels for training features generation selected from bone and skin annotations are called samples. There are three types of samples: (1) positive samples that represent bone, (2) positive samples that represent skin and (3) negative samples that represent neither bone nor skin.

**Figure 3:**
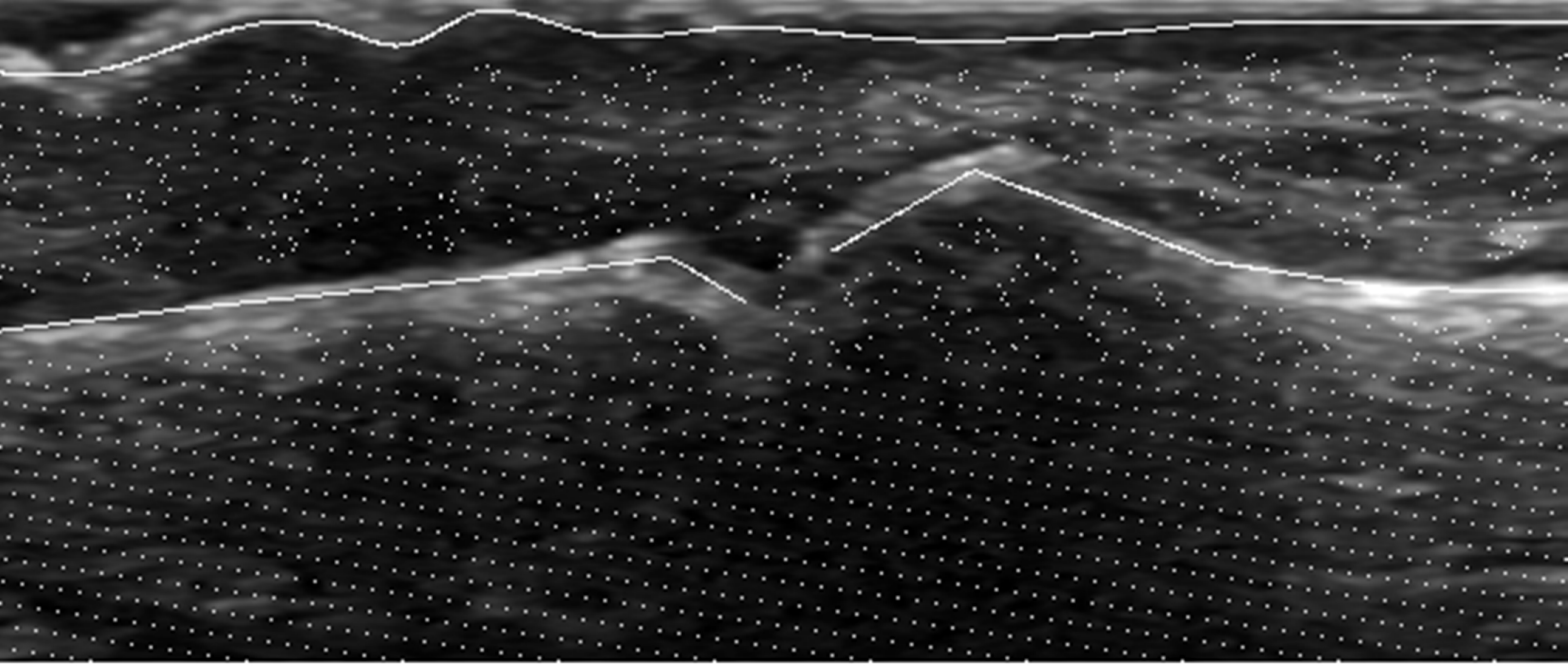
Example of pixel selection for samples generation.

The pixels used for generation of positive bone samples are the same as bone annotation points. Similarly, the pixels used for positive skin samples generation are the same as skin annotation points. By contrast, the pixels used for negative samples generation are selected from areas of an image that are neither bone nor skin regions. The bone and skin regions are defined as areas within a given radius from bone and skin annotation points. Thus, each pixel selected for the negative sample must lie in distance bigger than given radius from any pixel belonging to bone or skin annotation. Figure 3 presents the example of pixels selection that is further used for samples generation.

To obtain the proper characterization of the selected pixels, their neighborhood should also be considered. This is done by taking as a pixel representation the subimage [2⋅*w* + 1, 1] centered at the pixel, where 2⋅*w* + 1 is a vertical window around the pixel. The representation vector of a pixel *p*_*i*_ is defined as a vector of pixels *K*_*i*_ = (*p*_*k*,*l*−*w*_, *p*_*k*,*l*−*w*+1_, …, *p*_*k*,*l*+*w*_), where *k* and *l* are the image coordinates of the pixel *p*_*i*_.

**Figure 4:**
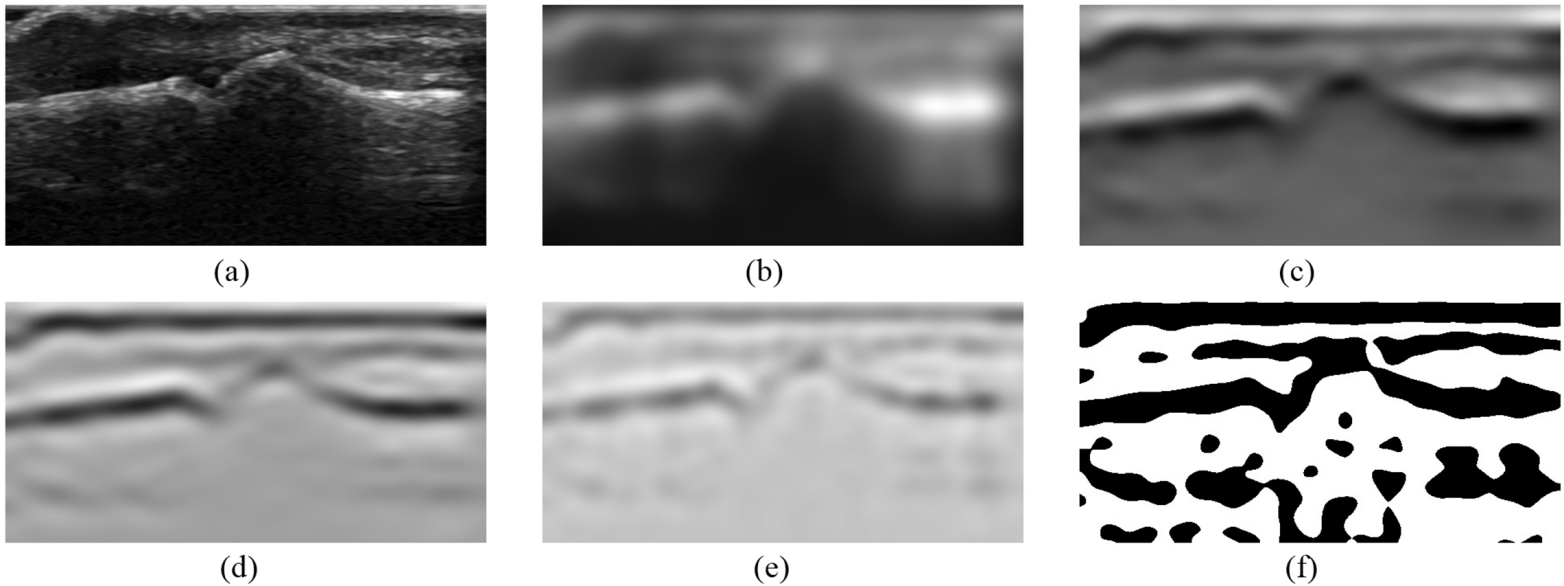
Images obtained from selected ITK filters: (A) the original image, (B) Gaussian smoothing, (C) smoothing first derivative, (D) smoothing second derivative, (E) Laplacian, (F) Laplacian positive threshold.

Once the pixels for samples are selected and their vertical neighborhood is defined the final samples generation process may start. First of all, a series of new images is obtained by use of different filters, where each new image is an output of particular filter applied to the original image. It includes Gaussian smoothing, first and second derivatives of Gaussian, Laplacian of Gaussian as well as thresholding filters. The Gaussian smoothing, as proposed in Deriche (1990), is done by recursive filtering that approximates an image convolution with the Gaussian kernels. The convolution with first and second derivatives of Gaussian is applied in vertical direction and the Laplacian of a Gaussian filter is obtained by applying the second derivative of a Gaussian in both vertical and horizontal directions. Additionally, all outputs of these filters are processed by the binary thresholding. This operation with the use of some given thresholds produces binary output images by mapping the original pixel values to black or white ones. All filtering operations use the efficient implementation of recursive filters (Deriche, 1993) taken from ITK library. The resulting images obtained by selected filters are presented in Fig. 4.

The set of images obtained by applying *n* filters to the original image *I* forms a vector *F* = (*I*_1_, *I*_2_, …, *I*_*n*_), which is used for final samples generation. The samples are generated by taking the values of all selected pixels with their vertical neighborhood *K*_*i*_ from each image *I*_*j*_ which is obtained from the *j*th filter. This generates for each *K*_*i*_ the matrix made from concatenated vectors of values extracted from filtering outputs *I*_*j*_ (where *j* ∈ [1, *n*]) for each pixel in the window, which can be denoted as:

(1) \begin{eqnarray*}{V}_{i}= \left( \begin{array}{@{}cccc@{}} \displaystyle {v}_{i11}&\displaystyle {v}_{i12}&\displaystyle ..&\displaystyle {v}_{i1n}\\ \displaystyle {v}_{i21}&\displaystyle {v}_{i22}&\displaystyle ..&\displaystyle {v}_{i2n}\\ \displaystyle :&\displaystyle :&\displaystyle :&\displaystyle :\\ \displaystyle {v}_{ik1}&\displaystyle {v}_{ik2}&\displaystyle ..&\displaystyle {v}_{ikn} \end{array} \right) .\end{eqnarray*}

**Figure 5:**
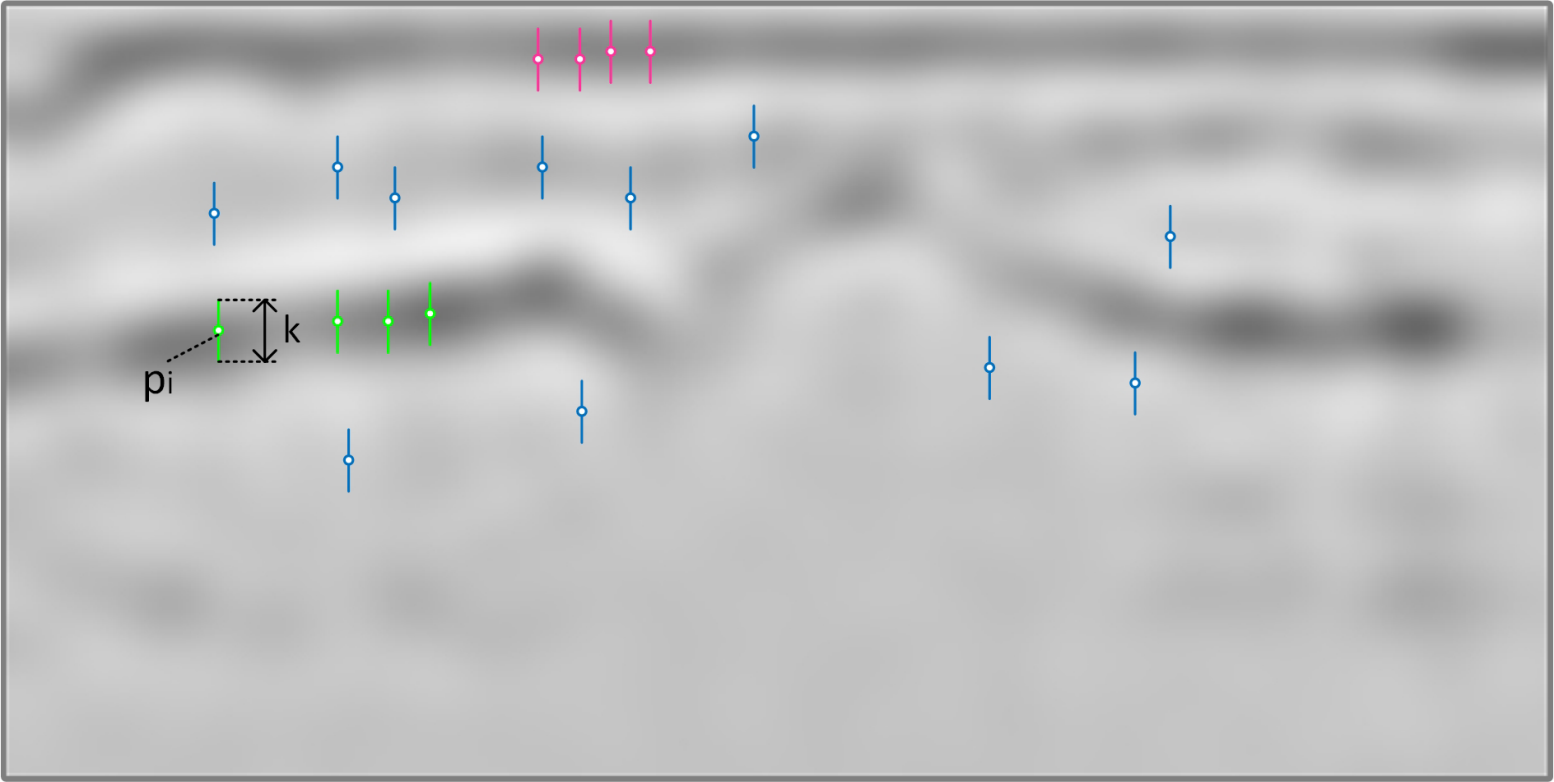
Different neighborhoods *K*_*i*_ taken from Laplacian filter output: green, pink and blue lines represent samples for bone, skin and none areas respectively.

The example of a few different neighborhoods *K*_*i*_ indicating the pixels to be taken from the Laplacian output as part of training samples is presented in Fig. 5.

The final training samples for given image *I* consist of three matrices. Each such matrix is a concatenation of matrices from Eq. (1) for all samples of given type *t*:

\begin{eqnarray*}& & {X}_{t}= \left( \begin{array}{@{}c@{}} \displaystyle {V}_{1},{V}_{2},..,{V}_{m} \end{array} \right) \end{eqnarray*}

(2) \begin{eqnarray*}& & = \left( \begin{array}{@{}cccc@{}} \displaystyle \left( \begin{array}{@{}cccc@{}} \displaystyle {v}_{111}&\displaystyle {v}_{112}&\displaystyle ..&\displaystyle {v}_{11n}\\ \displaystyle {v}_{121}&\displaystyle {v}_{122}&\displaystyle ..&\displaystyle {v}_{12n}\\ \displaystyle :&\displaystyle :&\displaystyle :&\displaystyle :\\ \displaystyle {v}_{1k1}&\displaystyle {v}_{1k2}&\displaystyle ..&\displaystyle {v}_{1kn} \end{array} \right) &\displaystyle \left( \begin{array}{@{}cccc@{}} \displaystyle {v}_{211}&\displaystyle {v}_{212}&\displaystyle ..&\displaystyle {v}_{21n}\\ \displaystyle {v}_{221}&\displaystyle {v}_{222}&\displaystyle ..&\displaystyle {v}_{22n}\\ \displaystyle :&\displaystyle :&\displaystyle :&\displaystyle :\\ \displaystyle {v}_{2k1}&\displaystyle {v}_{2k2}&\displaystyle ..&\displaystyle {v}_{2kn} \end{array} \right) &\displaystyle ..&\displaystyle \left( \begin{array}{@{}cccc@{}} \displaystyle {v}_{m11}&\displaystyle {v}_{m12}&\displaystyle ..&\displaystyle {v}_{m1n}\\ \displaystyle {v}_{m21}&\displaystyle {v}_{m22}&\displaystyle ..&\displaystyle {v}_{m2n}\\ \displaystyle :&\displaystyle :&\displaystyle :&\displaystyle :\\ \displaystyle {v}_{mk1}&\displaystyle {v}_{mk2}&\displaystyle ..&\displaystyle {v}_{mkn} \end{array} \right) \end{array} \right) \end{eqnarray*}

where *m* is a number of samples of given type, *t* ∈ {*Skin*, *Bone*, *None*}, *k* is a window size and *n* is a number of filters applied to the image *I*. Each column of matrix Eq. (2) represents the output values of particular filter for one neighborhood *K*_*i*_ of pixel *p*_*i*_ and each row represents the output values from all filters for one index in the neighborhood window *K*_*i*_ across all pixels *p*_*i*_. Such form of training data is directly passed as feature vectors to learning algorithms in the second phase of the processing described in following sections.

### Features for classification

Once the learning algorithms are trained on the feature vectors described in the previous chapter, they require a similar form of data for classification instead of raw pixel values from the original test image. The process of samples generation for classification is the same as for training samples generation. The only difference is that the samples are generated for all pixels *p*_*i*_ of the test image instead of those representing only positive and negative samples. It means that if the test image resolution is *M* × *N*, the number of pixels selected for samples generation is *M*⋅*N*. The final data for classification are also concatenated in the same way as the training data Eq. (2) using all vertical neighborhoods *K*_*i*_ and output values from all filters *F*, which gives the final size of the generated matrix as *M*⋅*N*⋅*n*⋅*k*.

## Learning and Classification

The learning part of the system was built using the scikit-learn environment, which offers many configurable algorithms suitable for three-class classification tasks. The list of evaluated classifiers is described below.

1. Gaussian Naive Bayes classifier (Zhang, 2004) is based on a conditional probability model defined by applying the Bayes’ theorem with strong (naive) assumption of independence between the features. The model parameters are estimated during training step with the use of maximum likelihood, and the classification rule relies on the most probable hypothesis selection according to the estimated probability distribution.
2. The k-Nearest Neighbour classifier (kNN) (Altman, 1992; Hastie, Tibshirani & Friedman, 2009) is one of the simplest classification methods which caches training samples at training step and then performs the classification for new sample by finding some predefined number of cached samples closest in distance to that new sample and using the label from them as a prediction.
3. Support Vector Machine (SVM) (Boser, Guyon & Vapnik, 1992; Chang & Lin, 2011) is a classifier which is trained to obtain a hyperplane splitting the samples in some sample space into different classes with margin maximization. The SVM classifier can use different kernels, which implicitly map the inputs into high-dimensional feature space and thus efficiently perform a non-linear classification. There were a few different kernels evaluated in this work including linear, polynomial and Radial Basis Function (RBF) kernel.
4. Decision Tree classifier (DTs) (Breiman et al., 1984; Hastie, Tibshirani & Friedman, 2009) is based on a directed graph of decisions inferred from the data features.
5. Random Forest classifier (Breiman, 2001) uses many decision trees classifiers executed on various subsets of samples averaging the results for improving accuracy and overfitting control. The Random Forest classifier belongs to ensemble method domain, where many predictions from basic classifiers are combined to improve generalizability and robustness.
6. Another ensemble method evaluated in this work is an AdaBoost classifier (Freund & Schapire, 1997; Zhu et al., 2009), which initially trains the basic classifier on the original dataset and after first evaluation it trains additional copies of the classifier on the same dataset but focusing on incorrectly classified cases during that evaluation.

Each classifier can be trained with training samples generated with use of ITK as described in previous sections. By using the three-class model for classifiers, they can learn simultaneously the classes representing two types of positive samples for bone and skin as well as negative samples for other parts of the image.

The classification is done by querying the particular classifier for the class of each pixel on the test image. The result of classification is written as a matrix of the same dimension as test image being classified and consists of the values indicating the class for a particular pixel, that is, bone or skin or none. The output matrix that represents detected skin and bone regions is then passed to the next phase of synovitis detection system, namely the part responsible for the approximation of clean lines for bone and skin, which are then used directly by the registration model (Segen, Kulbacki & Wereszczyński, 2015).

## Searching Parameter Space

**Figure 6:**
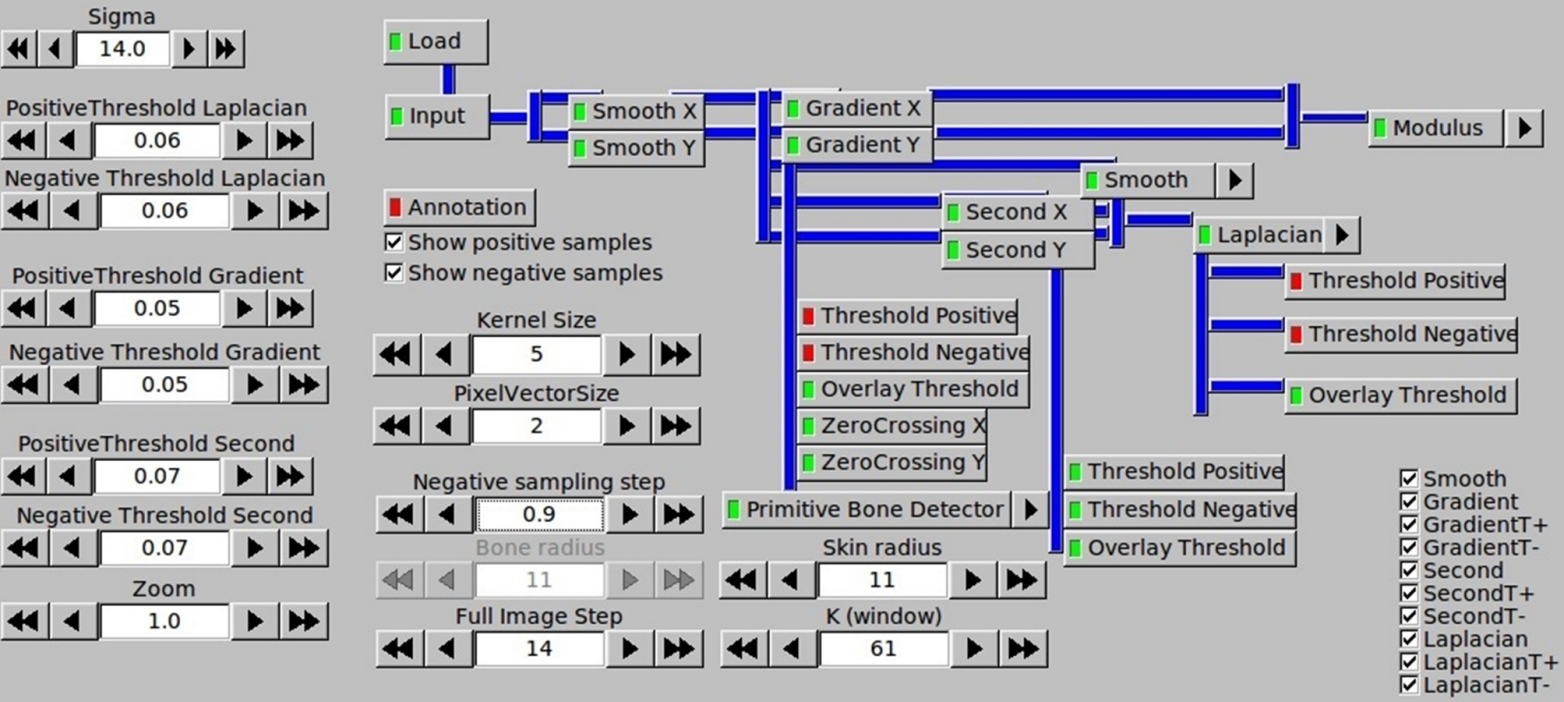
Configuration of samples generation by the ITK parameters panel.

Both the ITK based samples extraction and the scikit-learn based classifier is set up with many parameters, which directly affect the final detection result. The screenshot of the ITK parameters configuration panel for samples generation including filtering configuration is presented in Fig. 6. Even more parameters can be defined for different classifiers in the scikit-learn part.

The optimal configuration of bone and skin detector that gives the best efficiency relies on finding the optimal values for all these parameters. The number of all possible parameters is too high to execute the exhaustive search in a reasonable time taking into consideration the relatively long time of samples generation and classifier learning. Instead, the heuristic approach is applied using an easy protocol to define some series of parameters in a form of an extendable tree structure and filtering the large number results efficiently. The process of searching the optimal system configuration is performed according to Algorithm 1.

 
________________________________________________________________________________ 
Algorithm 1 Searching the optimal parameters of detector 
________________________________________________________________________________ 
  1: Define the parameters of the detection system for optimization, e.g.:  Z = 
     (z1,z2,z3,z4,z5) 
  2: Define the range of values for each parameter, e.g.: (a11,a12,a13) for z1 and 
     (a21,a22) for z2 
  3: Split Z into L groups G = {Z1,..,ZL}, e.g.: Z1 = (z1,z2), Z2 = (z4,z5) 
  4: Define the set of fixed parameters Fixed = {∅} 
  5: goto TREE(Z1) 
  6: TREE(Zi): 
  7: Generate the search tree from Zi in such way that each value of preceding 
     parameters is a parent for all defined values of its successive parameters, 
     e.g.   the branches bi  of tree for Z1  are following:  b1  =  (a11,a21),b2  = 
     (a11,a22),b3 = (a12,a21),b4 = (a12,a22),b5 = (a13,a21),b6 = (a13,a22) 
  8: Check each configuration of the system defined by params = Fixed ∪ bi 
     including feature extraction, learning and classifier performance evaluation 
  9: Select the branch b∗ from line 7 which gives the best evaluation result in 
     line 8 on the base of precision, recall, fallout and accuracy values 
 10: Fixed ← Fixed ∪ b∗ 
 11: G ← G ∖{Zi} 
 12: if G ⁄= ∅ then 
 13:      goto TREE(Zi+1) 
 14: else 
 15:      Exit 
________________________________________________________________________________    

Above iterative process can be repeated for a different split of parameters (line 3 in Algorithm 1) or by using additional parameters until some global criteria for evaluation result are met.

One of the most important uses of such iterative search is to find the best image filter combination for samples generation which is called a *feature selection*. It was proven during experiments that different filter sets give very different results: some filters have more impact on results than others and in some cases the smaller set of filters gives a better result than the bigger one. Some examples of different results for different filter combinations are presented in following section describing empirical results.

## Results

A set of 34 images was used for evaluation, which allowed obtaining around 120,000 samples including around 40,000 positive samples representing bone and skin and around 80,000 negative samples. We assumed that a test set of the size of 50,000 samples is adequate to assess the quality of the results. The remaining 70,000 samples are used for the training set, giving the training to test ratio of 60:40. Four metrics were used to evaluate the quality of predictions done by classifiers described in previous sections: precision, recall, fallout and accuracy. The results of classifier prediction are divided into four groups defined by the indicators listed below:

1. True Positives (TP) is a number of correct predictions of positive samples.
2. False Positives (FP) is a number of incorrect predictions of positive samples.
3. True Negatives (TN) is a number of correct predictions of negative samples.
4. False Negatives (FN) is a number of incorrect predictions of negative samples.

Above indicators are used to define the appropriate evaluation metrics (Zhu, 2004) according to a mathematical formulation in Eqs. (3)–(6).

Precision: indicates how many predictions of positive samples were correct.
  (3) \begin{eqnarray*}Precision= \frac{TP}{TP+FP} \end{eqnarray*}
Recall: indicates how many positive samples were correctly done among all positive samples and measures the sensitivity of classifier.
  (4) \begin{eqnarray*}Recall= \frac{TP}{TP+FN} \end{eqnarray*}
Fallout: indicates the proportion of incorrectly predicted positive samples to all negative samples and thus measures the classifier proneness to false detections.
  (5) \begin{eqnarray*}Fallout= \frac{FP}{FP+TN} \end{eqnarray*}
Accuracy: is the proportion of correct predictions out of all predictions performed by the classifier and measures the overall performance of a classifier.
  (6) \begin{eqnarray*}Accuracy= \frac{TP+TN}{TP+FP+TN+FN} .\end{eqnarray*}

The results achieved in the experiments are summarized in Table 1. Both learning and prediction time durations were measured only during the real activity of the classifier, which means that the file loading and feature preprocessing time is not included in the reported results. The prediction time refers to the prediction of a single test image. Each entry in Table 1 is an average result obtained from two learning rounds, where each round splits the image dataset into two exclusive subsets (training and test set) in a different way.

**Table 1:** Results from experiments.

Classifier	Precision	Recall	Fallout	Accuracy	Learn time	Detection time
Naive Bayesian	0.58	0.96	0.35	0.75	1 s	<1 s
kNN	0.85	0.78	0.06	0.88	30 s	2 min 10 s
SVM (linear)	0.94	0.91	0.03	0.95	7 h 45 min	33 s
SVM (polynomial)	0.92	0.86	0.04	0.93	2 days	16 s
SVM(RBF)	0.90	0.36	0.02	0.77	3 h	3 min
Decision trees	0.92	0.74	0.03	0.89	2 min	<1 s
**Random forest**	**0.96**	**0.79**	**0.02**	**0.92**	35 s	<1 s
Adaboost	0.70	0.84	0.17	0.83	11 min	<1 s

**Figure 7:**
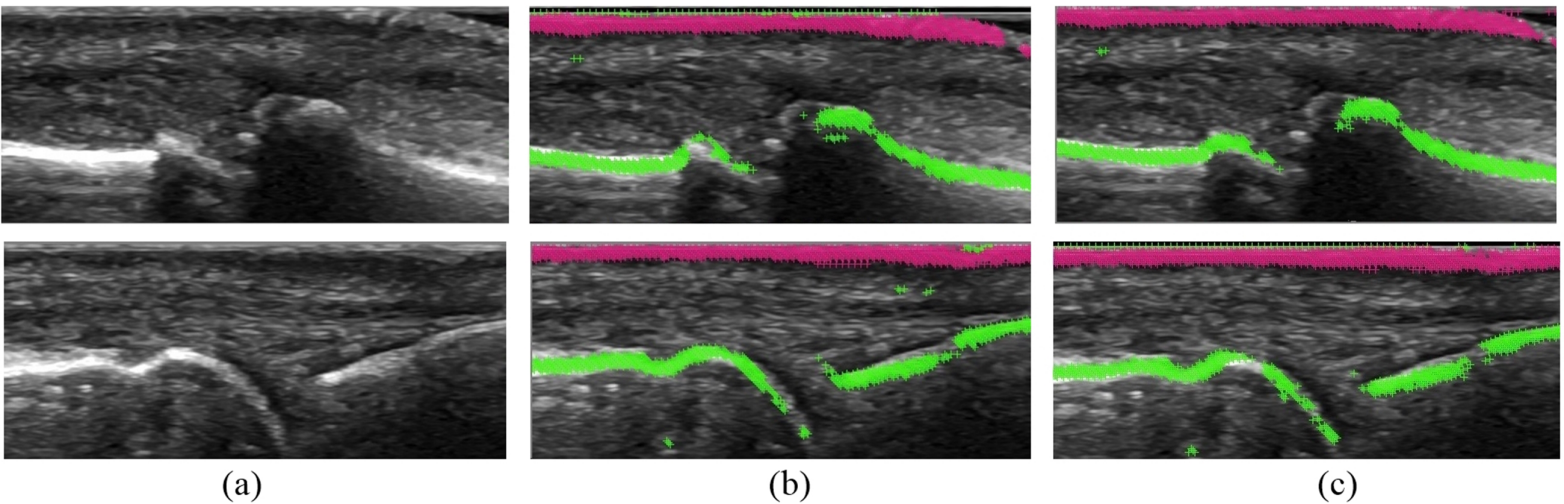
The results from experiments: (A) input images for detection, (B) features generated by optimal set of filters, (C) features generated by full set of filters.

The best results were achieved for the Random Forest classifier and SVM with polynomial and linear kernels giving the accuracy of 0.92, 0.93 and 0.95 respectively. Though SVM classifiers achieved the best overall accuracy, the Random Forest classifier was selected for the final use as it obtained the best precision and showed less proneness to false positive detection (*Fallout*), which gives less noise for further steps of the synovitis detection process. Additionally, the Random Forest classifier provides the best computational efficiency. Some examples of visualization results for the best configuration of the system are presented in Fig. 7.

**Figure 8:**
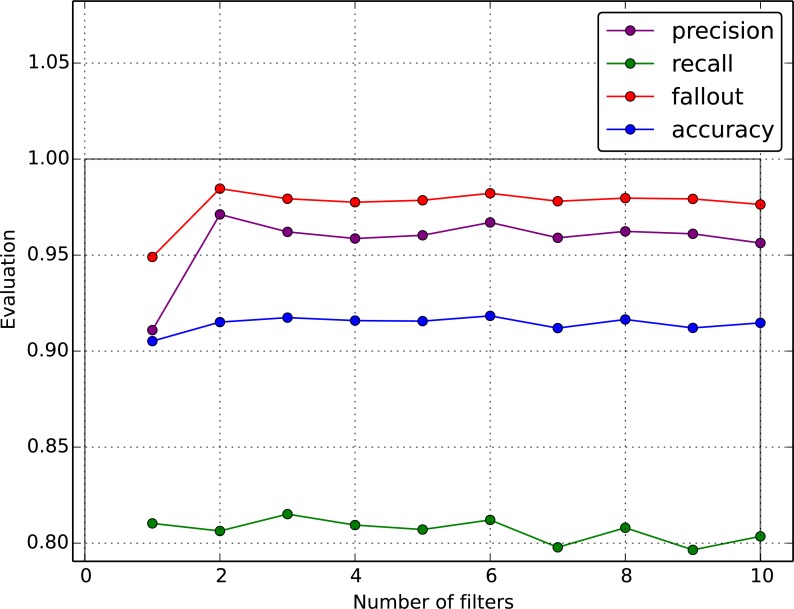
The results for the different filter combinations.

As mentioned in the previous sections, the selection of proper filter combination used for samples generation has a significant impact on the final result. The optimal configuration of filters that was found by our automatic heuristic search consists of Gaussian smoothing, first and second derivatives as well as Laplacian where the first and second derivatives have the most impact on the result. Adding the threshold filters makes the results to get worse. Figure 8 presents the example of samples generation where just a few filters gave a better result than the full set of filters.

## Conclusions

This paper presents a bone and skin detector which is an important part of a synovitis detection system (Medusa, 2015). The detector output is further processed by a registration method which improves the detection result, therefore the precision achieved by the bone and skin detector does not need to be perfect. The results presented in this paper are sufficient for the detector to fulfill its role as determined in tests that will be described in a future article. The optimization of bone and skin detector is done locally, based only on the comparison of results with the annotations. There is a plan to introduce a global feedback, from the output of the entire synovitis detector to find the best configuration of bone and skin detector for which the system is the most effective.

## Additional Information and Declarations

### Data Availability

The following information was supplied regarding data availability: Kulbacki, Marek (2018): bone-and-skin-detector-train-data.zip. figshare. 10.6084/m9.figshare.5881288.v1.
